# New steps of robot-assisted radical prostatectomy using the extraperitoneal approach: a propensity-score matched comparison between extraperitoneal and transperitoneal approach in Japanese patients

**DOI:** 10.1186/s12894-017-0298-z

**Published:** 2017-11-21

**Authors:** Satoshi Kurokawa, Yukihiro Umemoto, Kentaro Mizuno, Atsushi Okada, Akihiro Nakane, Hidenori Nishio, Shuzo Hamamoto, Ryosuke Ando, Noriyasu Kawai, Keiichi Tozawa, Yutaro Hayashi, Takahiro Yasui

**Affiliations:** 1 Department of Urology, Nagoya Tokushukai General Hospital, 2-52, Kouzouji-cho-kita, Kasugai, 487-0016 Japan; 2 Department of Nephro-urology, Nagoya City University Graduate School of Medical Sciences, 1, Kawasumi, Mizuho-cho, Mizuho-ku, Nagoya, 467-8601 Japan

**Keywords:** Robot-assisted radical prostatectomy, Extraperitoneal approach, Transperitoneal approach, Small physique, Trendelenburg position, Inguinal hernia, Propensity score

## Abstract

**Background:**

Robot-assisted radical prostatectomy (RARP) is commonly performed using the transperitoneal (TP) approach with six trocars over an 8-cm distance in the steep Trendelenburg position. In this study, we investigated the feasibility and the benefit of using the extraperitoneal (EP) approach with six trocars over a 4-cm distance in a flat or 5° Trendelenburg position. We also introduced four new steps to the surgical procedure and compared the surgical results and complications between the EP and TP approach using propensity score matching.

**Methods:**

Between August 2012 and August 2016, 200 consecutive patients without any physical restrictions underwent RARP with the EP approach in a less than 5° Trendelenburg position, and 428 consecutive patients underwent RARP with the TP approach in a steep Trendelenburg position. Four new steps to RARP using the EP approach were developed: 1) arranging six trocars; 2) creating the EP space using laparoscopic forceps; 3) holding the separated prostate in the EP space outside the robotic view; and 4) preventing a postoperative inguinal hernia. Clinicopathological results and complications were compared between the EP and TP approaches using propensity score matching. Propensity scores were calculated for each patient using multivariate logistic regression based on the preoperative covariates.

**Results:**

All 200 patients safely underwent RARP using the EP approach. The mean volume of estimated blood loss and duration of indwelling urethral catheter use were significantly lower with the EP approach than the TP approach (139.9 vs 184.9 mL, *p* = 0.03 and 5.6 vs 7.7 days, *p* < 0.01, respectively). No significant differences in the positive surgical margin were observed. None of the patients developed an inguinal hernia postoperatively after we introduced this technique.

**Conclusions:**

The EP approach to RARP was safely performed regardless of patient physique or contraindications to a steep Trendelenburg position. Our method, which involved using the EP approach to perform RARP, can decrease the amount of perioperative blood loss, the duration of indwelling urethral catheter use, and the incidence of postoperative inguinal hernia development.

## Background

Robot-assisted radical prostatectomy (RARP) has been used worldwide since it was introduced in 2000 [1, 2]. The 4-armed da Vinci® S surgical system (Intuitive Surgical, Sunnyvale, CA, USA), which is responsible for the widespread use of RARP, has 8-cm-wide arms, and the trocars should be spaced at intervals >8 cm to prevent them from colliding with each other [3]. RARP is commonly performed using the transperitoneal (TP) approach because it offers enough arm distance, a larger working space, and familiar laparoscopic intraperitoneal landmarks [2]. The working space is relatively smaller when using the extraperitoneal (EP) approach. However, the procedure can be performed with little effect on patients with previous intraabdominal surgery or severe obesity [4], and it causes minimal intraabdominal complications [2, 5]. Furthermore, because the steep Trendelenburg position is not required, the EP approach is effective in patients with contraindications to this position [6, 7]. However, the EP approach has several limitations including a small working space and collision of the robotic arms with one another. Within a small operating cavity, the separated prostate impedes the operator’s visibility, and performing the vesicourethral anastomosis becomes difficult. It is challenging to acquire enough space to keep the removed prostate out of the robotic view and avoid collision of the robotic arms in patients who are physically small in particular. The development of new methods is necessary to overcome these difficulties.

Herein, we introduce four new steps for the surgical procedure and investigate the feasibility of using the EP approach with six trocars over a 4-cm distance in a less than 5° Trendelenburg position. We also compare the surgical results and complications of RARP using the EP and TP approaches.

## Methods

The study protocol was approved by the Tokushukai Group Ethical Committee (approval number: TGE00700–016). Clinical data were gathered starting from the beginning of our experience with RARP. The database included preoperative, operative, and postoperative information.

From August 2012 to August 2016, we retrospectively reviewed the data of 200 consecutive patients who underwent RARP with the EP approach in a flat or 5° Trendelenburg position and 428 consecutive patients who underwent RARP with the TP approach in a steep Trendelenburg position. Our surgical team began performing RARP using the TP approach in May 2011. Prior to that, they had experience performing >600 laparoscopic radical prostatectomies using TP and EP approaches [8–11]. After we managed 135 cases of RARP with the TP approach, we began performing RARP with the EP approach.

### Preoperative, operative, and postoperative data

Preoperative clinical data pertaining to patient background and prostate cancer such as the prostate-specific antigen (PSA) value, Gleason score, and clinical T stage were collected. Patient background included age, physique (height, weight, and body mass index [BMI]), and medical history. Patients with glaucoma, severe valvular heart disease, or intracranial diseases (e.g., an unruptured cerebral aneurysm) were submitted a priori to RARP using the EP approach in advance to avoid complications from the Trendelenburg position. Operative characteristics including the total operative time, robot console time, vesicourethral anastomosis time, estimated volume of blood loss, weight of the removed prostate, performance of lymph-node dissection, and surgical complications were also studied. Pathological variables included the Gleason score, presence of extension, seminal vesicle invasion, and surgical margin status. Postoperative information including the duration of indwelling urethral catheters and presence of postoperative complications was collected. Functional outcome was assessed based on urinary continence 6 months postoperatively. Continence was defined as using no pads or one safety pad per day.

### Surgical procedure

All the surgeries were conducted by the same surgical team using the da Vinci® S surgical system. All surgeons had reached the plateau in the learning curve having had experience with more than 50 cases [12, 13]. RARP using the TP approach was performed as described in previous articles [14, 15]. RARP using the EP approach was performed as follows.

**Fig. 1:**
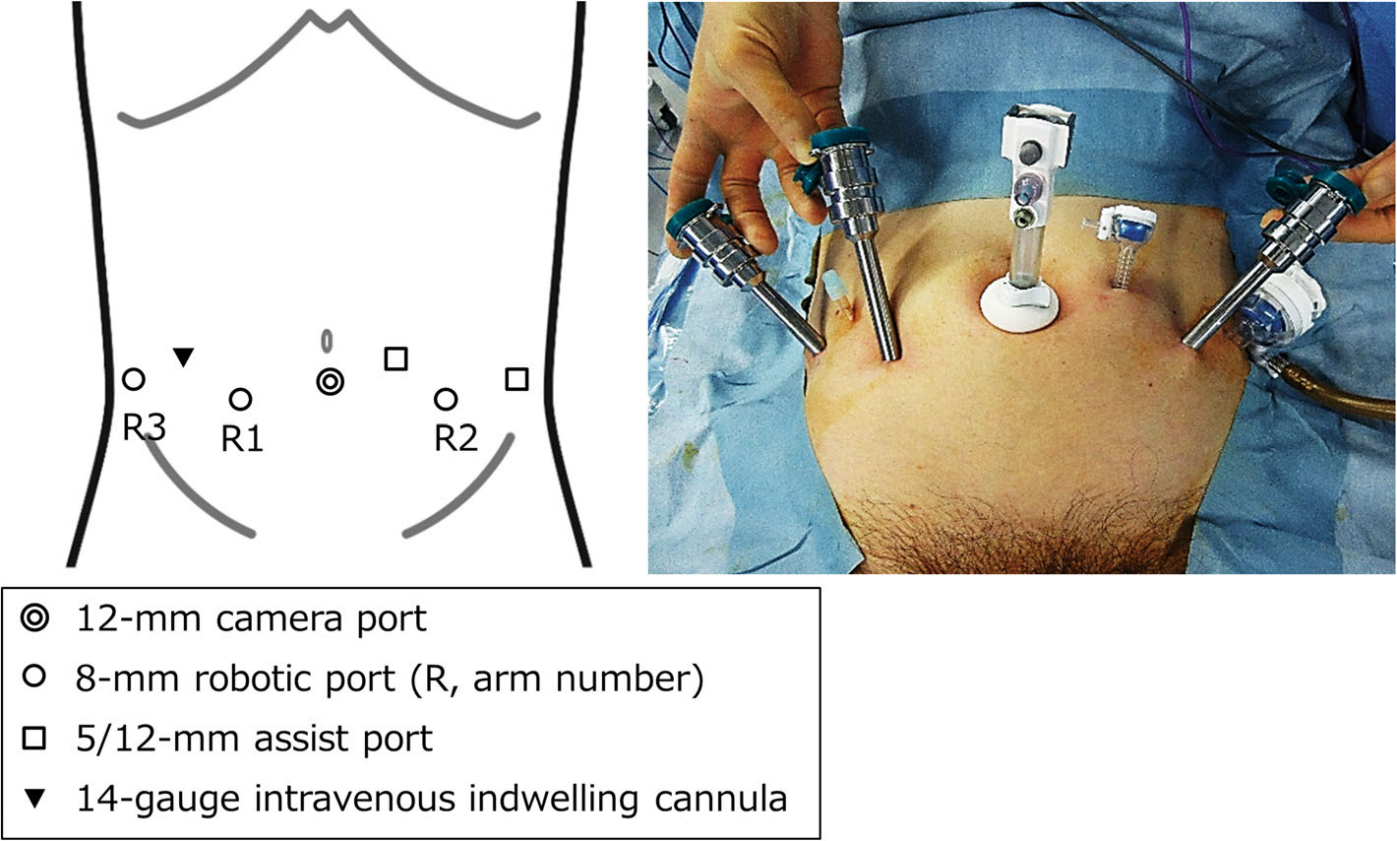
Placement of ports in the extraperitoneal approach to robot-assisted radical prostatectomy. In addition to six trocars, we place a 14-gauge intravenous indwelling cannula in the right lower abdominal region

First, we determined the position of the six trocars whose distances were 4 cm apart when performing RARP using the EP approach (Fig. 1). Traditionally, trocars are placed 7–8 cm apart during RARP using the EP approach [16–18]. However, it was difficult to space the trocars >7 cm apart in patients with small physiques. The decision to space the trocars 4 cm apart was based on a recommendation for performing robotic surgery in children [19].

**Fig. 2:**
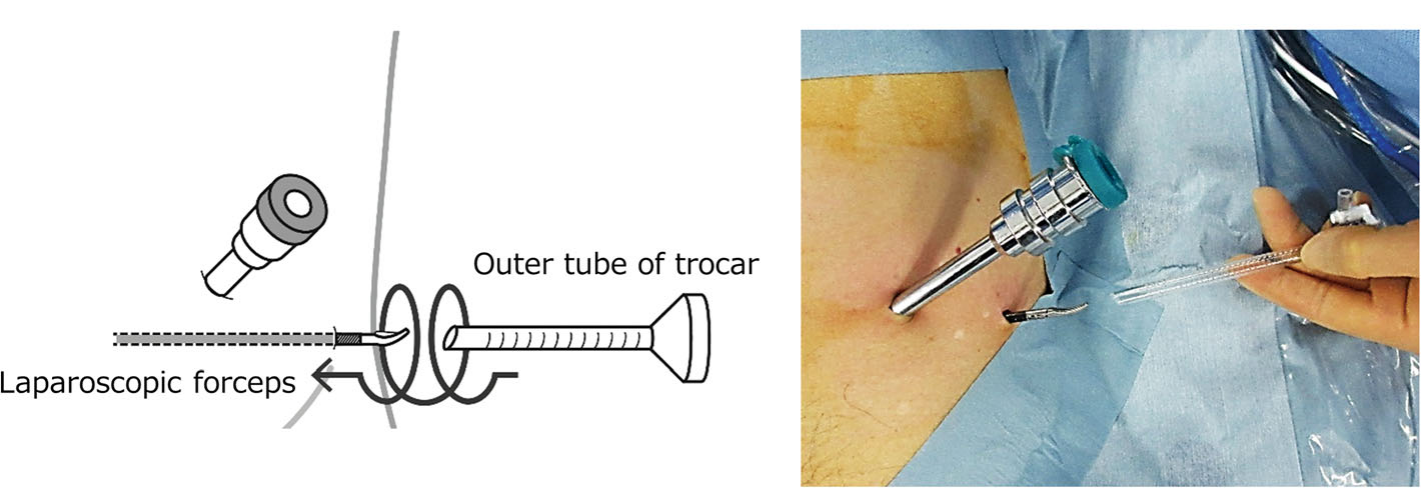
Method of placing a trocar in a small space (left side, assist port). We use laparoscopic forceps (a curved dissector) to penetrate from the small space of the extraperitoneal cavity to outside the body. Forceps are used instead of the inner cylinder of the trocar and only the outer tube of the trocar guides insertion of the forceps

Second, we created the EP space using a balloon dilator, finger assistance, and laparoscopic forceps. Furthermore, we developed a new technique for placing the trocar in a small space. A 4-cm transverse infraumbilical incision was made through the anterior rectus sheath. We intended to dissect the EP space digitally at the position of the trocars of robotic arm no. 1 or no. 2. We inserted a balloon dilator (Pajunk® balloon systems, Pajunk, Germany) and created the EP space using the laparoscopic view. If the EP space was insufficient to insert all six trocars, it was expanded using laparoscopic forceps through the trocar of robotic arm no. 1 or no. 2 as a working port. When placing a trocar in a small adhesive space, there is a risk for the trocar to become lost in the adhesion tissue. Therefore, we used a laparoscopic dissector with a pointed tip to penetrate the abdominal wall from the small space of the EP cavity to the outside of the body (Fig. 2). The shaft of the laparoscopy forceps was placed along the inner cylinder, and it was possible to extracorporeally guide the outer tube of the 5-mm trocar. When attempting to guide the 12-mm trocar, the tract of the trocar was gradually dilated after the 5-mm trocar was inserted, followed by the insertion of the 8-mm and 12-mm trocars.

**Fig. 3:**
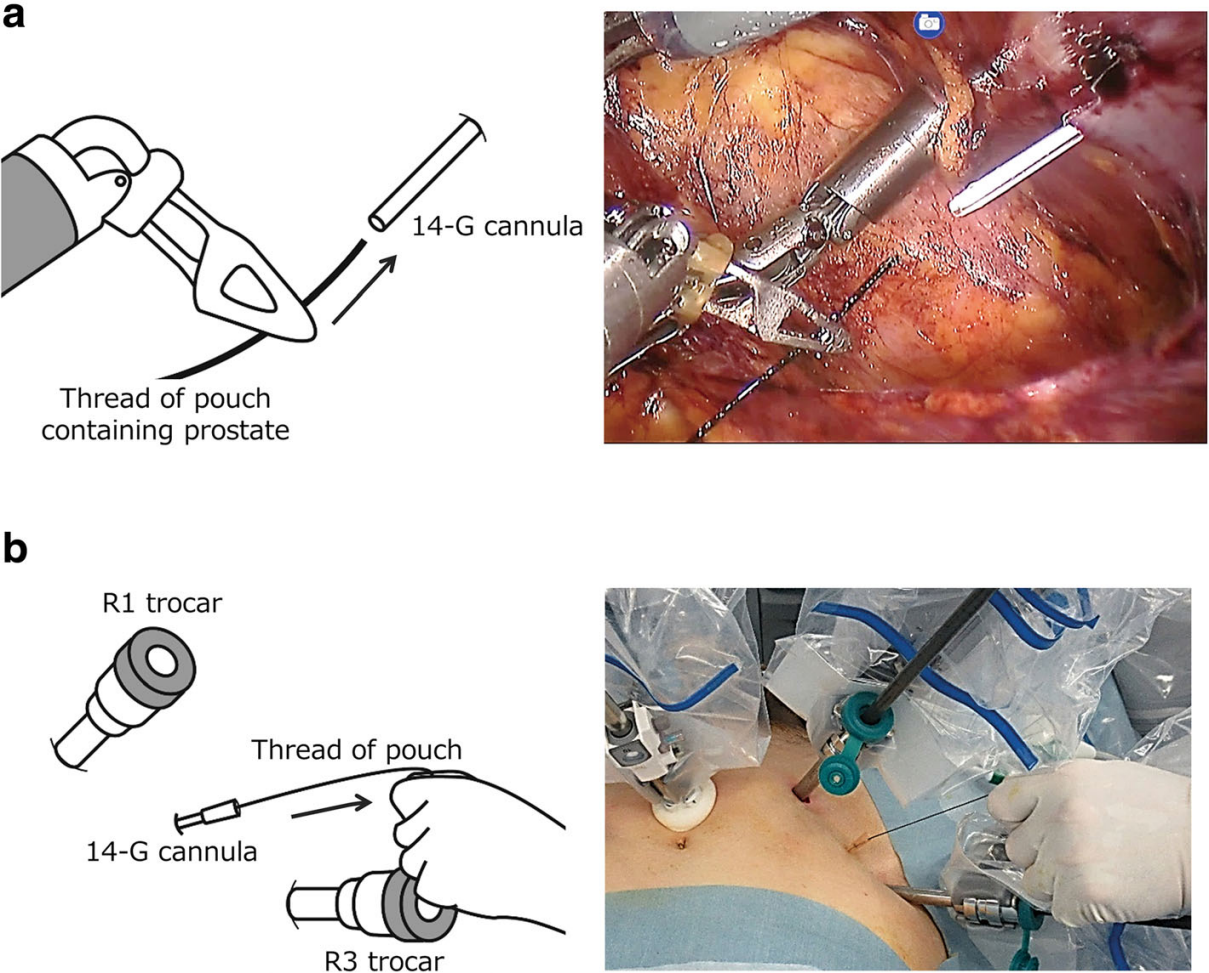
The prostate is placed in a pouch and retracted to the lower right abdomen. The thread of the pouch containing the prostate is pulled out of the body through the 14-gauge intravenous indwelling cannula (**a-b**) G, gauge

Third, we prepared to hold the freed prostate in the right abdominal EP space outside of robotic view. A 14-gauge intravenous indwelling needle was penetrated into the EP space from outside of the abdominal wall between the no. 1 and no. 3 robotic trocars (Fig. 1). After inserting a 14-gauge needle, the plastic cannula (outer cylinder) was left and the inner time (metal needle) removed. The plastic cannula was closed with the lid of the three-way stopcock to prevent carbon dioxide gas from leaking. After the prostate was dissected from the bladder neck in an antegrade manner, the separated prostate was stored in a pouch. Then, the thread of the pouch was pulled outside of the body through a 14-gauge plastic cannula (Fig. 3). The prostate was held on the right side of the abdomen outside of robotic view. After using the Rocco suture method, vesicourethral anastomosis was performed using the single-knot method with Lapra-Ty clips [9, 20].

**Fig. 4:**
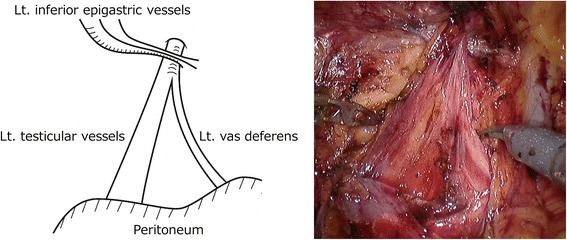
Technique to prevent postoperative inguinal hernia (Lt side). The peritoneum is dissected and peeled from the spermatic cord on the cranial side of the inner inguinal ring. Cord-like structures between the spermatic cord and peritoneum are transected, and the spermatic cord is separated into the testicular vessels and vas deferens Lt, left

Fourth, to prevent an indirect inguinal hernia postoperatively, we dissected and peeled the peritoneum from the spermatic cord at the inner inguinal ring. This technique was originally performed during retropubic radical prostatectomy [21], and it was introduced in RARP using the EP approach for all patients after the twenty-first patient. The cord-like structures of the cremaster muscle and fibrous tissue were transected around the spermatic cord, and the testicular vessels and vas deference were separated (Fig. 4).

Finally, the prostate was removed, and a 10-French closed drain was indwelled. The drain was removed on postoperative day 2, and on postoperative days 4–7, the urethral catheter was removed after confirming that there was no leakage using cystourethrography.

In selected patients whose risk of lymph node metastases was >10% according to the preoperative nomogram for Japanese patients or who were classified into the high-risk group according to the D’Amico criteria, pelvic lymph-node dissection was performed after the fourth step of preventing an inguinal hernia [22, 23].

### Propensity score matching and statistical analysis

Propensity scores were calculated for each patient using multivariate logistic regression analysis based on the preoperative covariates. The covariates used to compare the EP and TP groups were: follow-up duration, age, height, weight, BMI, prostate-specific antigen value, biopsy Gleason score, and clinical T stage. The covariates of the chronological comparison in the EP group excluded follow-up duration. Subsequently, balance of matching was assessed using statistical comparison. In propensity-score matched groups of the EP and TP approach, we analyzed operative results and postoperative outcomes. In the two propensity-score matched chronological subgroups in the EP group, operative and postoperative outcomes were also compared. The comparisons were evaluated using an χ^2^-test for qualitative variables and the Mann-Whitney U test for quantitative variables. A two-tailed *p*-value <0.05 was considered significant. Statistical analysis was performed with the JMP 13.0® software (SAS Institute Corp., Cary, NC, USA).

## Results

Patients in the EP and TP group were balanced using propensity score matching (Table 1). The patient with the smallest physique in the EP group was 143.6 cm tall and weighed 34.8 kg; he underwent RARP using the EP approach safely. Patient backgrounds such as follow-up duration, age, height, weight, and BMI were not significantly different between the groups (Table 1). Preoperative clinical characteristics such as the mean PSA level, biopsy Gleason score, clinical stage, and previous abdominal surgery, were also not significantly different between the groups (Table 1).

**Table 1:** Patients’ preoperative clinical characteristics by surgical procedures

Variables	EP approach	TP approach	*p-*Value
Patients (n)	190	190	
Follow-up (months)	27.3 ± 13.8 (4–52)	27.8 ± 154.3 (4–52)	0.78
Age (years)	69.4 ± 6.0 (51–82)	69.3 ± 4.7 (51–79)	0.70
Height (cm)	165.2 ± 6.1 (143.6–186.0)	165.4 ± 5.3 (152.5–183.2)	0.70
Weight (kg)	63.7 ± 9.3 (34.8–103.0)	63.6 ± 9.4 (38.0–122.0)	0.89
BMI (kg/m^2^)	23.3 ± 3.0 (16.9–34.5)	23.2 ± 2.9 (15.8–36.3)	0.73
PSA (ng/mL)	10.7 ± 11.6 (2.2–84.4)	10.8 ± 10.4 (2.3–73.1)	0.24
Biopsy Gleason score (%)			0.12
2–6	36 (19.0)	44 (23.2)	
7	100 (52.6)	80 (42.1)	
8–10	54 (28.4)	66 (34.7)	
Clinical stage (%)			0.15
T1	41 (21.6)	49 (25.8)	
T2	142 (74.7)	126 (66.3)	
T3	7 (3.7)	15 (7.9)	
Previous abdominal surgery (%)	62 (32.3)	65 (35.4)	0.91

*EP* extraperitoneal, *TP* transperitoneal, *BMI* body mass index, *PSA* prostate-specific antigen

Table 2 shows the operative and postoperative results, as well as complications in both groups. The mean operative time was significantly longer in the EP group than in the TP group (254.5 vs 225.8 min, *p* < 0.01). The mean robot console time and vesicourethral anastomosis time were not significantly different between the EP and TP group (180.5 vs 175.4 min, *p* = 0.07; and 30.3 vs 28.0 min, *p* = 0.09, respectively). The mean volume of estimated blood loss and duration of indwelling urethral catheters were significantly lower in the EP group than in the TP group (139.9 vs 184.9 mL, *p* < 0.01; and 5.6 vs 7.7 days, p < 0.01, respectively). There were no significant differences between the groups in terms of the positive surgical margin, lymph-node dissection rate, and urinary continence. The rate of postoperative inguinal hernia was significantly lower in the EP group than in the TP group (1.1 vs 7.4%, p < 0.01). There were no cases of conversion to open surgery in the EP group.

**Table 2:** Comparison of operative and postoperative results, and complications between the extraperitoneal and transperitoneal approaches

Variables	EP approach	TP approach	*p*–Value
Patients (n)	190	190	
Operative time (min)	254.5 ± 42.5 (144–464)	225.8 ± 44.0 (131–410)	<0.01
Robot console time (min)	180.5 ± 31.7 (98–304)	175.4 ± 40.9 (98–351)	0.07
Anastomosis time (min)	30.3 ± 11.7 (7–73)	28.0 ± 10.0 (8–66)	0.09
Blood loss (mL)	139.9 ± 118.7 (5–800)	184.9 ± 195.8 (0–1485)	0.03
Prostate weight (g)	46.0 ± 13.1 (22–96)	44.8 ± 16.4 (8–132)	0.13
Indwelling urethral catheter (days)	5.6 ± 1.7 (4–16)	7.7 ± 3.7 (4–33)	<0.01
Pathological Gleason score (%)			0.02
2–6	24 (12.6)	21 (11.1)	
7	118 (62.1)	142 (74.7)	
8–10	54 (28.4)	27 (14.2)	
Pathological stage (%)			<0.01
pT2	121 (63.7)	153 (80.5)	
pT3	69 (36.3)	37 (19.5)	
Positive surgical margin (%)			
pT2	13 (10.7)	30 (19.6)	0.09
pT3	37 (53.6)	21 (56.8)	0.76
Lymphadenectomy (%)	63 (33.2)	71 (37.4)	0.22
Continence (%)	173 (91.1)	170 (89.5)	0.60
Complications (%)			
Anastomosis stenosis	2 (1.1)	5 (2.6)	0.20
Blood transfusion	0 (0.0)	1 (0.5)	0.32
Colorectal injury	0 (0.0)	2 (1.1)	0.16
Conversion to open surgery	0 (0.0)	1 (0.5)	0.32
Ileus	0 (0.0)	2 (1.1)	0.16
Indirect inguinal hernia	2 (1.1)	14 (7.4)	<0.01
Symptomatic lymphocele	0 (0.0)	1 (0.5)	0.32

*EP* extraperitoneal, *TP* transperitoneal

Table 3 compares the surgical and pathological results of the 200 patients in the EP group subdivided into two chronological groups of 100 patients. The mean operative time, robot console time, and vesicourethral anastomosis time were significantly shorter in the latter group (257.9 vs 247.2 min, p < 0.01; 184.5 vs 174.1 min, p < 0.01; and 33.4 vs 24.2 min, p < 0.01, respectively). None of the patients developed an indirect inguinal hernia postoperatively after our method was introduced.

**Table 3:** Comparison of outcomes of the extraperitoneal approach according to the extent of surgical experience

	EP approach		
Variables	1–100	101–200	*p*–Value
Patients (n)	82	82	
Operative time (min)	258.6 ± 40.5	244.1 ± 43.2	<0.01
Robot console time (min)	183.5 ± 30.9	169.9 ± 26.2	<0.01
Anastomosis time (min)	33.1 ± 10.5	22.9 ± 7.7	<0.01
Blood loss (mL)	157.3 ± 116.6	104.8 ± 95.7	<0.01
Prostate weight (g)	46.3 ± 11.6	46.7 ± 14.4	0.93
Indwelling urethral catheter (days)	5.2 ± 1.0	5.6 ± 1.6	0.20
Pathological Gleason score (%)			0.04
2–6	14 (17.1)	4 (4.9)	
7	48 (58.5)	55 (67.1)	
8–10	20 (24.4)	23 (28.0)	
Pathological stage (%)			0.34
pT2	53 (64.6)	47 (57.3)	
pT3	29 (35.4)	35 (42.7)	
Positive srgical margin (%)			
pT2	6 (11.3)	2 (4.3)	0.15
pT3	20 (69.0)	12 (34.3)	<0.01
Lymphadenectomy (%)	26 (31.7)	31 (37.8)	0.25
Continence (%)	73 (89.0)	76 (92.7)	0.42
Complications (%)			
Indirect inguinal hernia	2 (2.4)	0 (0.0)	0.16
Anastomosis stenosis	1 (1.2)	1 (1.2)	1.00

*EP* extraperitoneal

## Discussion

Our method of RARP using the EP approach can be safely implemented, even in patients with small physiques. RARP using the EP approach has the benefit of reducing the amount of blood loss and shortening the duration of indwelling urethral catheter use compared with RARP using the TP approach.

We divided the surgical procedure into four steps. First, we arranged six trocars spaced 4 cm apart. In all 200 patients in the EP group, it was possible to perform surgery without revising the trocar placement. This result suggests that it is possible to perform RARP with the EP approach using six trocars spaced 4 cm apart.

Second, we created the EP space and guided the trocar with a new technique. Normally, the trocar is placed using finger assistance or laparoscopy to confirm the tip of the trocar so that it does not damage the surrounding structures [24]. However, when the location of trocar placement cannot be sufficiently exposed because of adhesions, it is difficult to appropriately confirm the tip of the trocar. Therefore, we used the laparoscopic dissector to penetrate from the EP space to outside of the body, to avoid the adhesive site, and to guide the trocar. This technique is a useful way to place the trocar into a narrow space, and it can be used during robotic surgery as well as any laparoscopic surgery.

Third, we developed a new technique to hold the separated prostate in the EP space. A 14-gauge cannula can serve as a tract for the thread of the pouch. During robotic or laparoscopic pyeloplasty, a 14-gauge cannula is used as a tract for the insertion of a ureteral stent [25, 26]. Thus, using an intravenous indwelling needle as a small tract through the abdominal wall can be helpful during minimally invasive surgery.

Fourth, the technique of preventing a postoperative inguinal hernia was simple and effective. This technique was originally performed during retropubic radical prostatectomy [21]. Before we introduced this technique, a postoperative inguinal hernia developed in 2 (10%) patients. However, none of the patients developed postoperative inguinal hernia after introducing this technique. The average follow-up duration in our study was over 27 months. The average interval between prostatectomy and postoperative inguinal hernia diagnosis was reported to be 10.6 months [21]. Thus, the follow-up duration we used in our study was sufficient to determine the effect of this technique.

Urethral anastomosis stenosis was observed in 1.1% of patients in the EP group and 2.6% of patients in the TP group. The incidence of strictures of the vesico-urethral anastomosis after radical prostatectomy has been reported to range from 0.5 to 32%, with most occurring within 5 months of radical prostatectomy [27–29]. Thus, the rate of anastomosis stenosis in our study was lower than those in the literature and the follow-up duration of our study was sufficient to evaluate the rate of anastomosis stenosis after RARP.

Symptomatic lymphocele occurred in 0% of patients in the EP group and 0.5% of patients in the TP group. We removed drains on postoperative day 2 after confirming a decrease in the drain fluid. The duration of pelvic drainage has been reported to influence the rate of symptomatic lymphocele [30]. The group whose drains were removed on postoperative day 1 exhibited higher rates of symptomatic lymphocele than patients whose drains were removed on postoperative day 7 and patients without drainage [30]. In general, lymphatic fluid cannot be absorbed by the peritoneal surface in the EP approach. Accordingly, the rate of lymphocele in the EP approach is greater than that in the TP approach. During the postoperative course of our study, we removed the pelvic drain after the amount of drain fluid decreased. Thus, the rate of lymphocele was low compared with that in the literature [31].

Our four new steps were demonstrated to be successful for performing RARP using the EP approach safely. The positive surgical margin and the time of operation, robot console, and vesicourethral anastomosis were compatible to those in the literature [32]. In particular, the amount of the blood loss and the incidence of the postoperative complications, such as inguinal hernia, anastomosis stenosis, and lymphocele, were low compared with those in the literature [31, 32].

The robot console time and vesicourethral anastomosis time of the EP approach became shorter after 100 cases, and they became shorter than those of the TP approach. However, the total operative time of the EP approach was still longer after 100 cases than that of the TP approach. There is a possibility that extra time was needed to create the EP space. As the operative space is relatively smaller in the EP approach, bleeding should be carefully controlled to maintain the robotic view. Vesicourethral anastomosis was performed after completing hemostasis. The values of blood loss and duration of indwelling urethral catheter use were significantly lower from the beginning of the EP approach than those of the TP approach. We first started RARP using the TP approach, and we overcame the learning curve of 50–100 cases before starting to perform the EP approach [12, 13]. However, 100 cases of the EP approach were needed to overcome the learning curve of EP approach.

We acknowledge that our study had strengths and weaknesses. One strength of our study is that we created a propensity-score matched comparison, which had a balanced covariate preoperative factor profiling, reducing the possibility of cofounding. Although our study was retrospectively designed, the propensity-score matched comparison might reduce selection bias. There were a few additional limitations to our study. First, the sample size might be relatively small for a study of this nature. Second, the model for propensity score matching may not include potentially relevant factors that are simply unknown at this time. Third, erectile data evaluation was not performed. Fourth, the operation was performed by the same surgical team but not by a single surgeon, which might have had an impact on the outcomes of the operation and the postoperative course.

## Conclusions

RARP with the EP approach using four new steps was safely performed regardless of patient physique and medical history. Our method of RARP with the EP approach can reduce the amount of perioperative blood loss, the duration of indwelling urethral catheters, and the incidence of postoperative inguinal hernia development. In addition to RARP using the TP approach, the EP approach is useful and it should be learned and mastered for all types of patients regardless of their medical history, if possible.

## Abbreviations

BMI: Body mass index
DVC: Deep vein complex
EP: Extraperitoneal
PSA: Prostate-specific antigen
RARP: Robot-assisted radical prostatectomy
TP: Transperitoneal
